# A Propagated Skeleton Approach to High Throughput Screening of Neurite Outgrowth for In Vitro Parkinson’s Disease Modelling

**DOI:** 10.3390/cells10040931

**Published:** 2021-04-17

**Authors:** Justus Schikora, Nina Kiwatrowski, Nils Förster, Leonie Selbach, Friederike Ostendorf, Frida Pallapies, Britta Hasse, Judith Metzdorf, Ralf Gold, Axel Mosig, Lars Tönges

**Affiliations:** 1 Department of Neurology, St. Josef-Hospital, Ruhr-University Bochum, 44803 Bochum, Germany; justus.schikora@rub.de (J.S.); friederike.ostendorf@rub.de (F.O.); britta.hasse@rub.de (B.H.); judith.metzdorf@ruhr-uni-bochum.de (J.M.); ralf.gold@ruhr-uni-bochum.de (R.G.); 2 Experimental Neurology, Center for Protein Diagnostics (ProDi), Ruhr-University Bochum, 44780 Bochum, Germany; 3 Bioinformatics Group, Department of Biology and Biotechnology, Ruhr-University Bochum, 44780 Bochum, Germany; Nina.Kiwatrowski@ruhr-uni-bochum.de (N.K.); nils.foerster-m9n@ruhr-uni-bochum.de (N.F.); leonie.selbach@ruhr-uni-bochum.de (L.S.); Axel.Mosig@ruhr-uni-bochum.de (A.M.); 4 Bioinformatics, Center for Protein Diagnostics (ProDi), Ruhr-University Bochum, 44780 Bochum, Germany; frida.pallapies@ruhr-uni-bochum.de

**Keywords:** Parkinson’s Disease, neurodegeneration, neurotoxicity, neurite outgrowth, neuronal morphology, high throughput screening, skeletonization

## Abstract

Neuronal models of neurodegenerative diseases such as Parkinson’s Disease (PD) are extensively studied in pathological and therapeutical research with neurite outgrowth being a core feature. Screening of neurite outgrowth enables characterization of various stimuli and therapeutic effects after lesion. In this study, we describe an autonomous computational assay for a high throughput skeletonization approach allowing for quantification of neurite outgrowth in large data sets from fluorescence microscopic imaging. Development and validation of the assay was conducted with differentiated SH-SY5Y cells and primary mesencephalic dopaminergic neurons (MDN) treated with the neurotoxic lesioning compound Rotenone. Results of manual annotation using NeuronJ and automated data were shown to correlate strongly ($R^{2}$-value 0.9077 for SH-SY5Y cells and $R^{2}$-value 0.9297 for MDN). Pooled linear regressions of results from SH-SY5Y cell image data could be integrated into an equation formula (y=0.5410·x+1792; y=0.8789·x+0.09191 for normalized results) with *y* depicting automated and *x* depicting manual data. This automated neurite length algorithm constitutes a valuable tool for modelling of neurite outgrowth that can be easily applied to evaluate therapeutic compounds with high throughput approaches.

## 1. Introduction

Parkinson’s Disease (PD) is a neurodegenerative movement disorder that is characterized by multifactorial cellular dysfunction of dopaminergic neurons [1]. Several impairments in cellular processes involved in protein homeostasis and mitochondrial function contribute to PD pathogenesis [2,3]. Abnormalities in protein aggregation, intracellular protein trafficking and protein disposal can result in α-Synuclein (aSYN) misfolding and aggregation as found in Lewy Bodies and Lewy Neurites in the human brain [3,4]. Pathological aSYN accumulation, impaired mitochondrial dynamics and high levels of reactive oxygen species cause dysfunction of mitochondria resulting in higher oxidative stress, which also contributes to neuronal death [2,5,6]. Neuroinflammation is another feature of PD pathology with important implications on neuronal health. Activated microglia cause neuronal death via different pathways. Release of proinflammatory cytokines, such as IL-1β, amplifies local inflammation and causes neuronal death [7,8,9]. By additional secretion of tumor necrosis factor (TNF) and activation of complement astrocytes are converted into neurotoxic phenotypes contributing to neuroinflammation [10,11,12]. Although there is much new evidence in PD research, the exact pathogenesis remains still unclear.

Due to the multifactorial character of PD an effective treatment strategy would have to target several pathological processes at a time. Therefore, approaches to prevent neuronal death [13,14,15], to replace neuronal cells [16] or to modulate inflammation and targeting aSYN have been followed [3,15]. While still no disease modifying approaches have shown to be effective, details of PD pathogenesis and of putative new therapeutic avenues are intensely studied.

To better analyze the multifactorial character of PD and other neurodegenerative diseases cellular analysis systems can be employed. Characterization of pathological events demands dissection of the complex PD pathogenesis into more simple molecular models enabling precise manipulation of contributing factors and introduction of therapeutic approaches. Differentiated dopaminergic cell models comprise immortalized cell lines (*SH-SY5Y*, *LUHMES*, *PC12*), primary dopaminergic neurons and induced pluripotent stem cells (iPSC) [17,18]. An advantage of the widely used SH-SY5Y cells is that they can be differentiated into neuronal-like cells exhibiting dopaminergic but also cholinergic or noradrenergic phenotypes [19,20]. Simple culturing techniques and cell amplification enable the generation of a large number of neuronal cells with dopaminergic phenotypes. These can be subjected to several neurotoxin-based screening experiments for PD, which are especially suitable for modelling effects of oxidative stress [19] using Rotenone, 1-methyl-4-phenylpyridinium (${\mathrm{MPP}}^{+}$), 6-hydroxydopamine (6-OHDA). Primary dopaminergic neurons are usually prepared from the ventral mesencephalon of mouse or rat embryos. They represent another model extensively studied in PD research and are often applied for analysis of molecular pathomechanisms [18].

In our study, we applied the pesticide Rotenone in different concentrations to induce neuronal damage to SH-SY5Y cells and MDN. Rotenone inhibits the complex I of the mitochondrial respiratory chain and thereby elicits oxidative stress and can promote the aggregation of alpha synuclein [21,22]. These induced molecular pathologies make Rotenone a valid and commonly used toxin in and PD models.

When screening for cellular pathologic events or therapeutic responses, neurite outgrowth represents an important readout. Aberrant neurite morphology is strongly associated with neurodegenerative disorders [23,24] and a critical determinant of neuronal connectivity that influences the processing and distribution of information within neural circuits [25]. Especially the morphological analysis of neuronal cultures and evaluation of morphologic changes is time consuming and laborious. Furthermore, it is in part dependent on subjective appreciation of neurite net features such as the detection of small neurite branches in objects with low fluorescence intensity or from fragmented neurites.

Small-scale screenings are mostly conducted by manual or semi-automated quantification of neurite outgrowth, for example using well-established tools such as NeuronJ [26]. It offers high accuracy but needs manually annotated start- and endpoints, which results in a time-consuming process. Currently available openly accessible approaches for high throughput screening utilize a variety of different approaches and platforms as summarized in Table 1. Additional algorithms are able to evaluate cultures with higher complexity like neuron-astrocyte cocultures [27] and whole neurospheres from iPSC [28,29,30]. This allows for disease modelling focused on cell type interaction. However, most of the existing approaches for now are limited to the analysis of single neurons or low-density neuron cultures. Yet, for screening highly complex neurite structures in medium or high throughput, new and more reliable approaches are required. Several studies [31,32,33] rely on commercial image analysis packages such as *Amira* (Visage Imaging), *HCA-Vision* (CSIRO Biotech Imaging), *Imaris* (Bitplane), *Cellomics* (Thermo Fisher Scientific), *GE IN Cell Analyzer* (GE Healthcare) and *Neurolucida* (MBF Bioscience), yet these packages are ‘turnkey’ solutions that lack the adaptability to novel complex cell culture conditions, and typically defy systematic validation and comparison [34].

At the same time, very recent novel approaches to morphological skeletonization [35,36] have not yet been utilized and validated in the context of analyzing neuronal morphology. Due to their rigorous algorithmic foundation and well-understood time complexity, it is a natural goal of our present contribution to establish these *propagated skeleton* approaches for analyzing the neurite structures in complex high-density neuron cultures.

We here present an approach using a skeletonization algorithm to evaluate neurite outgrowth and additional morphologic features on image data of the SH-SY5Y neuronal cell line and MDN. The presented approach allows for quantification of neurite outgrowth in a fully automated and high throughput manner with no manual input required after starting the pipeline. We demonstrate that this approach shows a strong correlation with quantification obtained from the well-established semi-automated neurite tracing method of NeuronJ. Furthermore, we were also able to extract other morphological features such as number of nuclei, nuclei size, soma size and the number of branches.

## 2. Materials and Methods

### 2.1. SH-SY5Y Cell Culture

The SH-SY5Y cell line was obtained from *DSMZ* (German Collection of Microorganisms and Cell Cultures GmbH, Braunschweig, Germany) and cultured in DMEM/Nutrient Mixture F-12 with GlutaMAX™ Supplement (DMEM/F-12, GlutaMAX™ Supplement; 31331028, Gibco^®^, Thermo Fisher Scientific Inc., Waltham, MA, USA) containing 10% heat-inactivated fetal bovine serum (FBS Standard, South America origin; P30-3306, PAN-biotech, Aidenbach, Germany) and 1% Penicillin-Streptomycin (Penicillin-Streptomycin (10,000 U/mL); 15140122, Gibco^®^, Thermo Fisher Scientific Inc.). Cells were cultivated at 37 ${}^{\circ }$C with 5% ${\mathrm{CO}}_{2}$ at saturated humidity. Morphology and behaviour were frequently monitored to sustain high culture standards.

### 2.2. SH-SY5Y Differentiation and Time Course of Experiments

The differentiation (as presented by [20] was carried out in 24-well plates with 12 mm coverslips coated first with poly-D-lysine (poly-D-lysine homobromide; 27964-99-4, Merck KGaA, Burlington, MA, USA) for minimum 24 h following a laminin coating (laminin from Engelbreth-Holm-Swarm murine sarcoma basement membrane; 114956-81-9, Merck KGaA) for at least 4 h. After incubation with 0.25% trypsin-EDTA (trypsin-EDTA (0.25%), phenol red; 2520056, Gibco^®^, Thermo Fisher Scientific Inc.), cells were counted and the number needed to achieve a density of 40,000 cells per well was added to the differentiation medium 1. This medium was DMEM, high glucose, GlutaMAX™ Supplement (DMEM, high glucose, GlutaMAX™ Supplement; 61965026, Gibco^®^, Thermo Fisher Scientific Inc.) with 5% heat-inactivated fetal bovine serum (FBS Standard; P30-3306, PAN-biotech), 1% Penicillin-Streptomycin (Penicillin-Streptomycin (10,000 U/mL); 15140122, Gibco^®^, Thermo Fisher Scientific Inc.) and 10 µM all-trans-retinoic acid (retinoic acid; R2625, Merck KGaA). The differentiation medium 2 contained Neurobasal™(Neurobasal™Medium; 21103049, Gibco^®^, Thermo Fisher Scientific Inc.) with additional 1% N-2 (N-2 Supplement (100X); 17502048, Gibco^®^, Thermo Fisher Scientific Inc.), 1% Penicillin-Streptomycin (Penicillin-Streptomycin (10,000 U/mL); 15140122, Gibco^®^, Thermo Fisher Scientific Inc.), 1% GlutaMAX™ (GlutaMAX™ Supplement; 35050061, Gibco^®^, Thermo Fisher Scientific Inc.) and *BDNF* (Recombinant Human/Murine/Rat BDNF; 450-02, PeproTech^®^, Rocky Hill, NJ, USA) to achieve a concentration of 10 µg/mL. After 3 days with differentiation medium 2 cells were incubated for 24 h with Rotenone (Rotenone; 83-79-4, Merck KGaA) diluted in dimethylsulfoxide (DMSO). Final Rotenone concentrations were 100 nM, 250 nM, 500 nM, 1000 nM, 2500 nM and 5000 nM.

### 2.3. In Vitro Assay of Primary Mesencephalig Dopaminergic Neurons MDN

For the preparation of MDN pregnant Sprague Dawley rats (Charles River Laboratories, Wilmington, MA, USA) were sacrificed on embryonic day 14. After isolation of embryonic midbrains, neuronal tissue was dissociated and neurons were seeded on poly-D-lysine (poly-D-lysine homobromide; 27964-99-4, Merck KGaA) cover slips. Using Neurobasal™(Neurobasal™Medium; 21103049, Gibco^®^, Thermo Fisher Scientific Inc.) with 1% B27 supplement (Life Technologies), 1% GlutaMAX™ (GlutaMAX™ Supplement; 35050061, Gibco^®^, Thermo Fisher Scientific Inc.), 1% Penicillin-Streptomycin (Penicillin-Streptomycin (10,000 U/mL); 15140122, Gibco^®^, Thermo Fisher Scientific Inc.) and 0.1% L-ascorbinacid 200 mM (Sigma-Aldrich, St. Louis, MO, USA) neurons were cultured for 5 days with half of the medium was changed on day 2. On day 3, cells were treated with Rotenone at final concentrations of 10 nM, 20 nM, 40 nM or 100 nM for 48 h. As described above, Rotenone was diluted in DMSO and for preperation in cell culture further diluted with neurobasal medium.

### 2.4. Immunocytochemistry

For immunofluorescence imaging SH-SY5Y cells grown on coverslips were fixed for 10 min with 4% paraformaldehyde in phosphate buffered saline (PBS) at pH 7.4. After washing with PBS with 0.1% triton (PBT 1) for 5 min three times, cells were blocked with PBT 1 for 20 min at room temperature. Primary antibodies, anti-neurofilament (anti-neurofilament 200 antibody produced in rabbit; N4142, Merck KGaA), were diluted in PBT 1 and incubated with samples for 1 h at roomtemperature. Next, cells were washed with in PBT 1 with bovine serum albumin (PBSA) for 5 min three times before being incubated with the secondary antibodies, *Alexa* 448 (Goat anti-Rabbit IgG (H+L) *Alexa* Flour 488; A-11008, Thermo Fisher Scientific Inc.), PBSA. Afterwards coverslips were washed with PBS for three times and mounted with $4^{\prime },6$-diamidino-2-phenylindole (DAPI) Flouromount (DAPI Flouromount-G^®^; 0100-20, SouthernBiotech, Birmingham, AL, USA).

MDN were fixed on day 5 with 4% of paraformaldehyde for 10 min at room temperature, washed three times with PBS, permeabilized with 0.1% triton (Sigma-Aldrich) in PBS for 5 min and were then blocked with 10% normal goat serum (Biozol, Eching, Germany) for 10 min at room temperature. Cells were incubated over night at 4 ${}^{\circ }$C with the primary antibody anti-tyrosine hydroxylase (1:1000, Merck Millipore, Burlington, MA, USA). After washing cells with PBS for 5 min three times, cells were incubated with secondary antibody, *Alexa* 448 (Goat anti-Rabbit IgG (H+L) *Alexa* Flour 488; A-11008, Thermo Fisher Scientific Inc.), for 45 min at 37 ${}^{\circ }$C. Afterwards coverslips were washed with PBS for three times and mounted with DAPI Flouromount (DAPI Flouromount-G^®^; 0100-20, SouthernBiotech).

**Figure 1:**
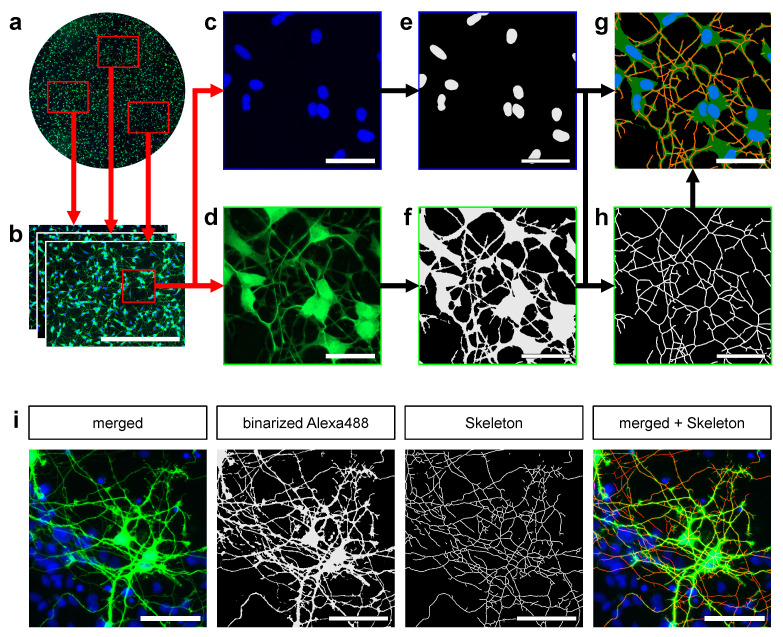
Fully automated skeletonization of neurite outgrowth. (**a**) Shows a representative image of a coverslip (*not to scale*) from which three images (**b**) (scale bar =500 µm) are added to the image data set. Each analyzed image spans 1376×1038 px (representing 884.89µm×667.52µm). For better visualization a representative section is shown in the following (**c**,**d**). Scale bars =50 µm. For analysis of neurite outgrowth and morpholgy *Alexa* 488 channel (**c**) and *DAPI* channel (**d**) are seperated. (**e**) shows the processed *DAPI* channel image as a binary image. In (**f**) the processed *Alexa* 488 channel is depicted, showing the neurite and soma area as a binary image. The *Alexa* 488 channel image is skeletonized. The full skeleton without soma substraction is shown in (**h**). For better visualization the skeleton was morphologically dilated. For visual evaluation the skeleton with soma substraction, the binarized *Alexa* 488 and *DAPI* channel image combined into one image (**g**). Representative images of MDN as they were used in the validation of the presented approach are shown in (**i**). First, the input image is depicted with a merged *Alexa* 488 and *DAPI* channel image. For both *Alexa* 488 images the binarized image and afterwards produced skeleton are shown. For better visualization the skeleton was morphologically dilated. By combining the final skeleton and the input image the effectiveness of the skeletonization can be evaluated.

Images were acquired on a fluorescence microscope (Olympus BX51, Germany) using a 20× objective (Olympus UPlanFl 20×, Japan) for SH-SY5Y cells and 40× objective (Olympus UPlanFl 40×, Japan) for MDN. *Alexa* 488 was excited with a 488 nm laser and detected with a 520/535 nm bandpass filter. DAPI was excited with a 405 nm laser and detected with a 450/50 nm bandpass filter. In experiments with SH-SY5Y cells 6 images were taken per condition. For analysis of the MDN 8 images were aquired per condition (Figure 1a,b). For development and validation of the algorithm a dataset consisting 528 images (288 from the SH-SY5Y cell assay and 240 for the assay of MDN) with additional images of different cell types used in development. Acquired images had the following properties: resolution 1376×1038 px, file format 16 bit TIFF (*tagged image file format*). Further image processing and analysis was carried out manually and automatically as described in the following.

### 2.5. Manual Ground Truth Measurement

**Figure 2:**
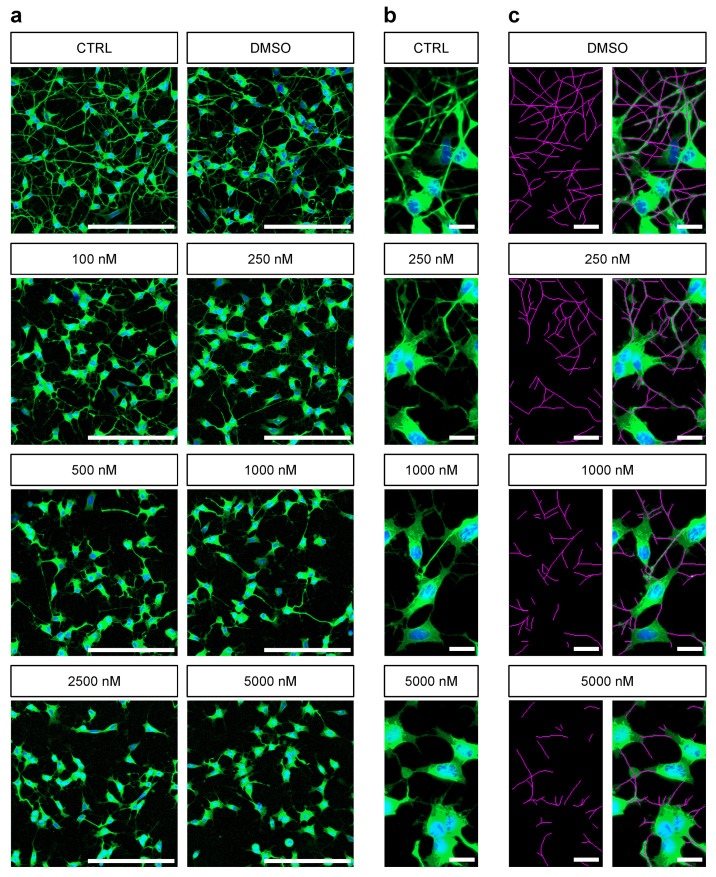
Dose-dependet effect of different Rotenone concentrations (100 nM, 250 nM, 500 nM, 1000 nM, 2500 nM and 5000 nM) on differentiated SH-SY5Y cells: (**a**) Representative micrographs of differentiated SH-SY5Y cells 24 h after treatment with Rotenone and control conditions. Stained against Neurofilament (green). Cell nuclei were stained with DAPI (blue). Scale bar =200 µm. (**b**) Cell morphology of differentiated SH-SY5Y cells in higher magnification. Stained against Neurofilament (green). Cell nuclei were stained with DAPI (blue). Scale bar =20 µm. (**c**) Exemplary annotation of neurite outgrowth performed manually with the ImageJ-Plug-In NeuronJ. Scale bar =20 µm.

Images were loaded in ImageJ and neurites were traced manually using the ImageJ-Plug-In NeuronJ (Figure 2c). The accumulative neurite length from all cells and the cell count of the image from the DAPI channel were used to calculate the relative neurite length. In addition to DAPI channel cell count, the number of TH${}^{+}$-cells was determined for images of MDN. All values were normalized to positive control and DMSO-treated solvent control (DMSO) for further evaluation.

### 2.6. Automated Image Processing

The algorithms and implementations in this work are designed to be run autonomously in a high throughput fashion. Practically, this means starting the pipeline once will evaluate a number of designated experiments with immunoflourescence image data successively without manual input or interruptions. The program will allocate resources dynamically and save results at runtime.

Figure 1 shows a general overview of the workflow. This section will describe the algorithms and image processing for every image acquired (see Section 2.4). These images are acquired and then split into their respective fluorescent color image channels (DAPI and Alexa488) each. Depending on the channel, the following algorithms are applied:

General Preprocessing. A number of image processing steps are taken to reduce noise and streamline image quality. In our assay, this translates to identifying several parameters that account for variations in staining artifacts and variations in light exposure levels. As such, brightness is dynamically reduced where necessary. The grayscale channel images are binarized using *Phansalkar* [41] local thresholding. To reduce noise, *morphological closing* is applied to the images and structures not matching a plausibility criterion (≤$82.71\;{µm}^{2}$) are removed.

DAPI Channel. The binary image mask obtained from this channel is used to determine nuclei area and count. Clumped or overexposed nuclei can be difficult to segment manually. This is often the case, when analyzing MDN. Thus, the *Watershed* algorithm [42] is used to separate clustered nuclei. The number and size of nuclei can be derived by separating isolated foreground contours using [43].

Alexa488 Channel. Depending on the compound and preprocessing, neurite growth can result in arborized structures that are too thin to be processed by the skeletonization. Yet these structures are vital to trace. Thus, to improve segmentation and skeletonization, the binary *Alexa* 488 channel image is scaled up by a factor of 3. To extract morphological features such as neurite length and number of branches, the skeletonization algorithm of Durix et al. (see Section 2.7) is applied. The precision parameter ϵ was set to 10 px, corresponding to 6.43 μm.

Combination of imaging channels. Our approach implemented the detection of a neuron’s soma and prevents skeletonization in soma areas. First off, by overlapping the nuclei masks and neurite masks, intersecting nuclei contours can be identified as neurons.

In a feedback process, every neurite contour not intersecting with a neuron is removed from the respective mask. This is done to remove false positives and staining artifacts. Another feedback process takes place during pruning (see Section 2.7). There, candidate skeleton branches are automatically disregarded if they overlap with the neuron mask. This is done to realistically reflect annotations and measure neurite lengths. Afterwards, statistics such as neurite length and branching points can be derived from the skeleton. By subtracting the neuron mask from the neurite mask, the neurite area is calculated.

### 2.7. Skeletonization and Pruning

The skeleton (or medial axis) of a shape is defined as the set of points that lie inside the shape and have more than one closest point on the boundary. Skeletonization has many applications in for example object recognition, image analysis and shape decomposition [44]. It is an important tool in biomedical imaging studies and often used for the morphological analysis of elongated structures such as vessels, pollen tubes and neurons [28,44,45,46].

Skeletons are known to be sensitive to noise in the boundary of an object, which results in spurious, uninformative branches. As this is a problem that often occurs in digital images, skeletonization methods include so-called pruning procedures, which remove the uninformative branches. Depending on the utilized pruning approach, the resulting skeletons may differ considerably. Thus, the choice of skeletonization method is important for the quality of results.

For the experiments, we utilized the algorithm of Durix et al. [35], which was specifically designed for discrete shapes. The skeleton is computed by propagating through the shape. Their pruning approach avoids propagating in directions of noise and therefore the creation of uninformative branches. This is determined by a precision parameter ϵ, which corresponds to the distance (in px, measured as Hausdorff distance) between the original shape and the approximated shape as represented by the skeleton. Small protrusions (smaller than 2×ϵ) are considered as uninformative and no branch is created.

During skeletonization the parameter ϵ affects the efficacy of automated neurite annotation. In this work, the key parameter ϵ of the propagated skeleton approach is set to 10 px (corresponding to 6.43 µm) resulting in a robust detection of neurites without excessive branching. Selection of ϵ depends on the morphological character of the respective cell type in particular on the neurite width.

### 2.8. Statistical Analysis and Validation

Statistical analyses were performed using GraphPad Prism version 8 (La Jolla, California). Dose-response curves for neurite toxicity, cell number, nucleus area, soma area and branching of SH-SY5Y cells were obtained from three, respectively, five independent experiments with six technical replicates per condition. The same values were evaluated on MDN with eight technical replicates per condition. Raw values from manual or automated analysis were first normalized either to cell count or to the number of TH${}^{+}$-cells obtained either by manual nucleus counting with ImageJ [47] or output data of the algorithm. Subsequently normalized values from each experiment were normalized to each positive control (CTRL) and DMSO-treated solvent control (DMSO). Data were further analyzed as percentage of solvent control and presented as percentage of solvent control ± standard error of the mean (SEM) if not described otherwise. Resulting mean values are averaged among all examined experiments. Significant differences within a dose–response relationship were analyzed using the analysis of variance (ANOVA) with an Ordinary one-way ANOVA (p<0.05). For dose–response curves ${\mathrm{LD}}_{50}$ were determined by using a sigmoidal dose–response fit (variable slope) fit with values normalized to DMSO-treated solvent control and values for solvent control as upper constraint. Differences between both methods were analyzed with multiple *t* testing. Analysis was conducted on results of every image, which was manually and automatically annotated with a paired *t* test assuming consistent standard deviations to determine if values were statistically different (p<0.05). For further comparison of intermethodological differences we performed simple linear regression with *y* depicting automated and *x* depicting manual data.

### 2.9. Implementation and Hardware

The pipeline proposed in this work is designed to run automatically and performs image analysis algorithms on fluorescence microscopy image batches of arbitrary size and count in a high throughput manner. The software is implemented in C++ version 14 for *Linux* and is using image analysis algorithms provided by *OpenCV* [48] and ImageJ. Since many evaluations are independent from each other, the pipeline is able to run evaluations concurrently. The pipeline also runs inside a *Docker* [49] version 19.03.13 image, making it more robust to running on different systems and computational environments. All evaluations were performed on a server, running *Ubuntu* 18.05.5 LTS with a 112 core CPU and 754 GB RAM.

## 3. Results

### 3.1. Manual Analysis of Rotenone-Induced Alterations of Neurite Outgrowth

**Figure 3:**
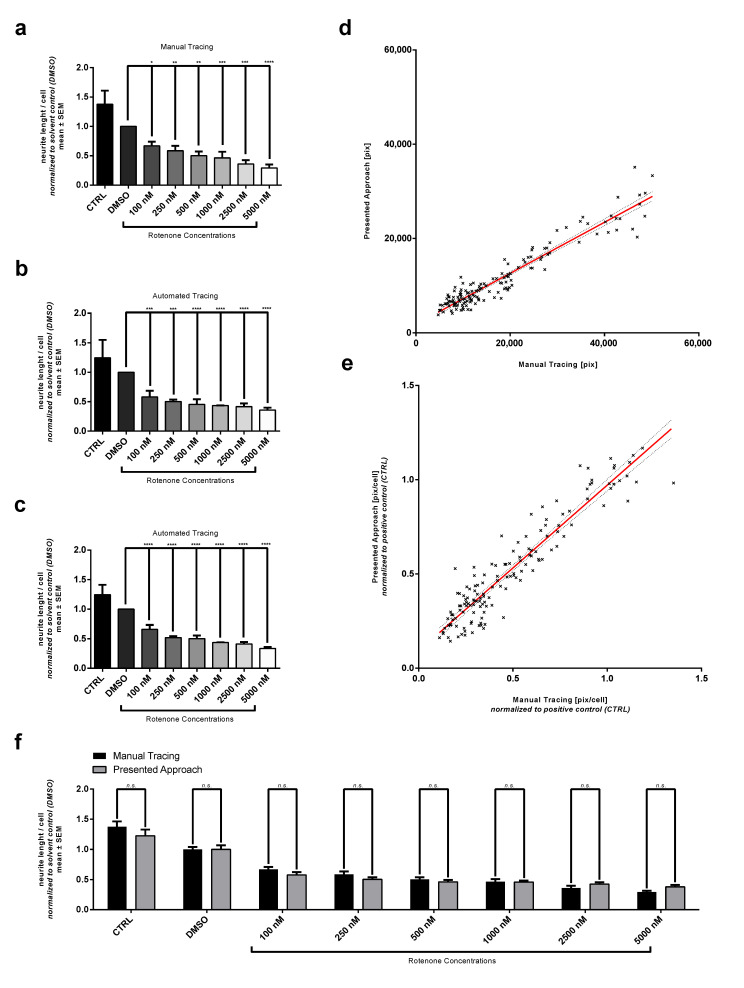
Comparison of results from automated and manual analysis. (**a**) Neurotoxic effect of Rotenone on the neurite network quantified with manual analysis: Mean neurite length per cell normalized to DMSO-treated solvent control (DMSO). (**b**) showing the neurotoxic effect of Rotenone on the neurite network quantified with automatically with our approach. Summed neurite length per image obtained from the output data was first normalized to cell count and afterwards normalized to DMSO. (**c**) showing results from automated analysis with two additional experiments. Data are shown as mean ± SM. * Significantly different (p<0.05) between mean of solvent control (DMSO) and mean of according Rotenone treatment condition. Respectively ** with p<0.01, *** with p<0.001 and **** with p<0.0001. (**d**) Linear regression performed on results of summed neurite per image quantified with automated and manual analysis. (y=0.5410·x+1792; $R^{2}=0.9077$) (**e**) Linear regression performed on results of neurite length normalized to cell count and afterwards to positive control (CTRL) (y=0.8789·x+0.09191; $R^{2}=0.8841$). (**f**) Mean values from automated analysis compared to mean values obtained from manual analysis. (*n.s.* with p>0.05).

We established an Rotenone lesioning model in order to generate different levels of impaired neurite outgrowth. Differentiated SH-SY5Y cells were treated with Rotenone concentrations ranging from 100 nM to 5000 nM, respectively, 5 µM. Cells in both control conditions positive (CTRL) and DMSO-treated solvent control (DMSO) developed a high-density neurite network. They distributed evenly and interconnected via these arborized neurites resulting in a high neurite density per cell (Figure 2a,b). Manual quantification of neurite length was first carried out with the ImageJ-Plug-In NeuronJ. We detected neurites in CTRL that had a length up to 345.6 µm while in DMSO they reached up to 333.4 µm in length. Rotenone treatment induced in a concentration-dependent effect on neurite outgrowth indicated by a decline in mean neurite length per cell. For better inter experiment comparison results were normalized to the DMSO-treated solvent control (DMSO) (Figure 3a). Rotenone treatment with concentrations of 100 nM and 250 nM obtained a moderate lesion of 33.2%±7.1% and 41.4%±8.4%. 500 nM Rotenone reduced the neurite length per cell to 50.2%±7.1% (absolute: 64.4 $\frac{µm}{\mathrm{cell}}$) compared to DMSO-treated solvent control (absolute: 127.3 $\frac{µm}{\mathrm{cell}}$). The highest Rotenone concentration (5000 nM) lead to a decrease in neurite length by 70.7%±5.8%. We also determined a mean ${\mathrm{LD}}_{50}$ value at 624.0 nM.

### 3.2. Automated Neurite Outgrowth Analysis of Rotenone Treated SH-SY5Y Cells

For the evaluation and comparison with manually generated neurite outgrowth quantification we developed a novel image processing pipeline. After initial histogram adjustment and executing a local threshold, binarized pictures were processed for better skeletonization efficacy. Skeletonization was executed using an algorithm proposed by Durix et al. [36]. We were able to automatically create masks representing the soma area of a cell with the module *distance transformation*. After subtracting somata from the skeleton, neurite length was calculated. The quantification of nuclei was carried out using *distance transformation* followed by a *Watershed* algorithm. The number of nuclei was derived by separating isolated foreground contours. Using the summed neurite length per image and matching cell count from the output data, the neurite length per cell was calculated and subsequently normalized to the DMSO control. As shown in Figure 3a a dose–response relationship was also detected when employing our approach. Because the complex arborization of neurites is challenging for the correct detection by the algorithm, we optimized preprocessing the skeletonization of neurites by applying histogram adjustment and noise reduction with *morphological closing* and therefore were able to maintain a high number of neurites after binarization.

Comparing the neurite length per cell from both manual and automated readouts, the initial automated results were 28.0% less than manual calculations. Mean neurite length per cell from automated analysis was found to be 107.0 $\frac{µm}{\mathrm{cell}}$ for CTRL and 93.9 $\frac{µm}{\mathrm{cell}}$ for DMSO. The skeletonization algorithm (Figure 1) showed reduced neurite measurements when analyzing binarized images because small neurites with low fluorescence intensity were not detected after preprocessing. However, with an increased the number of additional automatically analyzed datasets, the comparability to the manually annotated results improved (Figure 3c). Treatment with 100 nM and 250 nM Rotenone led to reduction in neurite length of 34.1%±7.3% or 48.2%±2.1%, respectively. In total, 500 nM Rotenone reduced the neurite length by a half to 50.1%±4.8% compared to DMSO control. Under treatment of 5000 nM Rotenone, neurite length was decreased by 66.4%±3.2%. In comparison, the manual analysis resulted in the following neurite length: 33.2%±7.1% (100 nM), 41.4%±8.4% (250 nM), 50.2%±7.1% (500 nM) and 70.7%±5.8% (5000 nM). ${\mathrm{LD}}_{50}$ from automated analysis yielded a value of 584.0 nM compared to ${\mathrm{LD}}_{50}$ of 624.0 nM from manual analysis as mentioned above (see Section 3.1).

### 3.3. Comparison of Automated and Manual Analysis Shows High Correlation

To validate the results generated by automated neurite length analysis, we performed a set of different tests. First, we compared results generated automatically and manually with linear regression applied on values from both readouts. The values of summed neurite length per image were compared to assess the efficacy of our skeletonization algorithm. When plotting results of all three experiments which were analyzed with both methods, a mean $R^{2}$-value of 0.9077 was calculated. The pooled linear regression results in the following equation: y=0.5410·x+1792 (y= automated; x= manual analysis) (Figure 3d).

Normalized linear regression with results pooled from all three experiments analyzed both with NeuronJ and the algorithm resulted in a $R^{2}$-value of 0.8841 and the following equation: y=0.8789·x+0.09191 (Figure 3e). No significant difference could be detected between both methods (Figure 3f).

The ability for high throughput screening using the presented approach is confirmed by a mean runtime of 2.72±1.1 min per image (mean runtime for SH-SY5Y cell image data with 1.68±0.4 min and mean runtime for MDN image data with 3.75±2.2 min).

### 3.4. Enhancement with In Vitro Detail Parameters from Automated Neuronal Analysis

Automated image analysis allows for quantification of additional parameters such as cell count based on DAPI nuclei signal. The binarization of DAPI-channel enables the calculation of total nucleus area or differentiation of apoptotic from healthy nuclei due to their morphological features. Cell somata of SH-SY5Y cells can be subtracted from the skeleton as a mask generated by a distance transformation. The total mask area can be quantified as total soma area and then be normalized to the cell count. Furthermore, the number of neurite branches can be determined.

**Figure 4:**
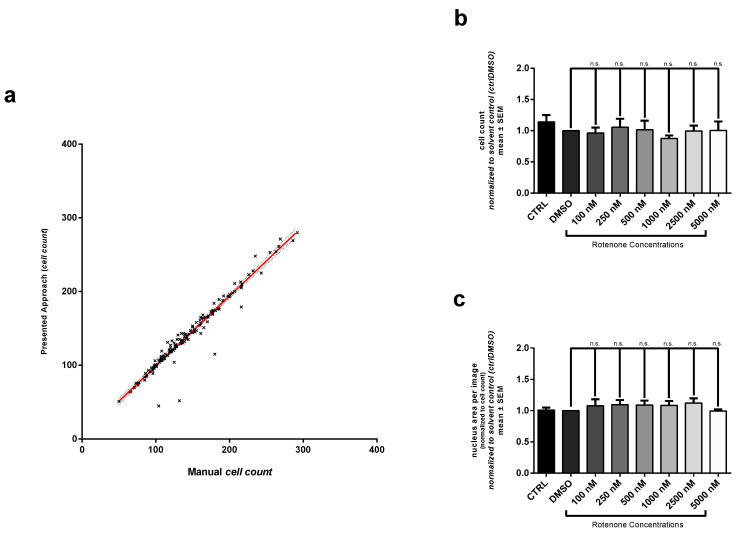

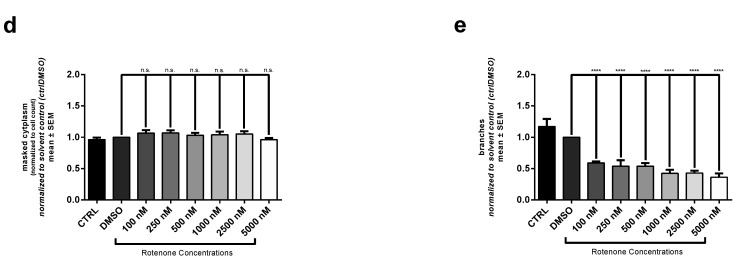
Additional morphological endpoints analyzed by our approach. (**a**) Linear regression performed on results automated and manual cell count. (y=0.9511·x+0.3678; $R^{2}=0.9430$) The following endpoints were quantified: (**b**) cell number, (**c**) nucleus area, (**d**) cell body area and (**e**) neurite arborization. For interpretation results from quantification of cell number and neurite arborization were normalized to solvent control (DMSO). Values of nucleus and cell body area were first normalized to cell count and afterwards normalized to solvent control (DMSO). Data are shown as mean ± SM. * Significantly different (p<0.05) between mean of solvent control (DMSO) and mean of according Rotenone treatment condition. Respectively ** with p<0.01, *** with p<0.001 and **** with p<0.0001.

In the analysis of the SH-SY5Y cell cultures, cell counts were not altered with different Rotenone dosages (Figure 4b). Quantification of the nucleus area or the cell body area also showed no dose dependent effect (mean nucleus are of 977.14±14.51 $\frac{{µm}^{2}}{\mathrm{cell}}$ and mean soma area of 1357.41±19.51 $\frac{{µm}^{2}}{\mathrm{cell}}$) for all conditions. No difference between DMSO-treated solvent control (DMSO) (mean nucleus area of 930.01±43.56 $\frac{{µm}^{2}}{\mathrm{cell}}$ and mean soma area of 1286.54±42.54 $\frac{{µm}^{2}}{\mathrm{cell}}$) and 5000 nM Rotenone (mean nucleus area of 924.35±62.02 $\frac{{µm}^{2}}{\mathrm{cell}}$ and mean soma area of 1277.26±53.17 $\frac{{µm}^{2}}{\mathrm{cell}}$) could be detected (Figure 4c,d). Furthermore, we extracted the number of neurite branches with our algorithm method. In contrast to neurite length, the number of branches per cell is more sensitive to Rotenone induced toxicity. Exposure to 100 nM Rotenone resulted in a reduction of 41.0±2.4 in the number of branches. With 250 nM and 500 nM the number of branches was decreased even stronger by 46.0±9.4% and 49.5±5.3%, respectively. The highest concentration of Rotenone (5000 nM) leads to a more pronounced decrease (63.8±6.1%) (Figure 4e).

### 3.5. Automated Neurite Outgrowth Quantification of Rotenone Treated MDN

**Figure 5:**
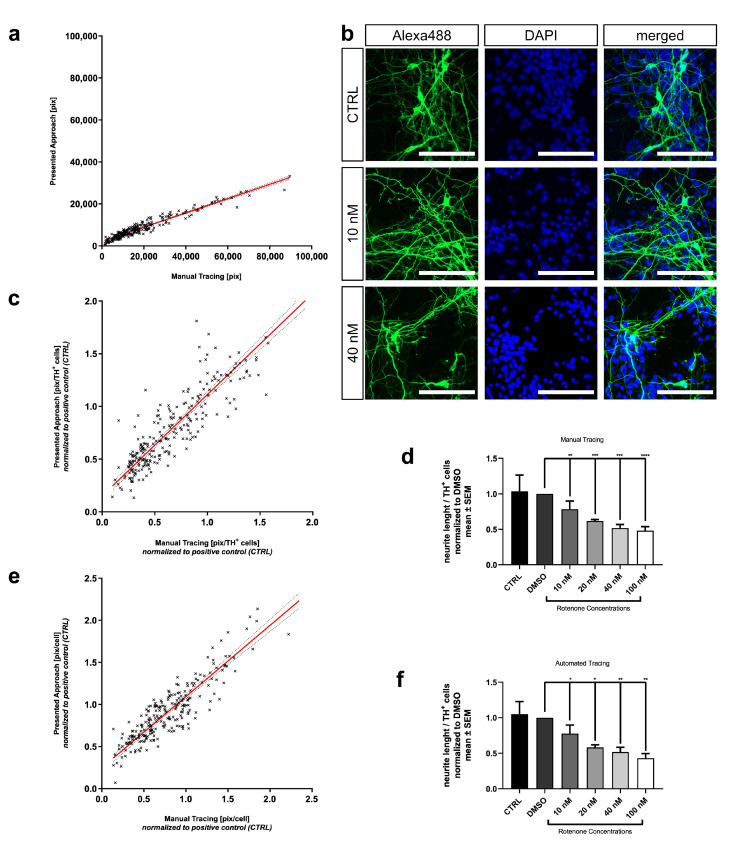
Further validation with MDN. (**a**) Linear regression performed on results of summed neurite length per image quantified with the presented approach and manual analysis using NeuronJ. (y=0.3403·x+2109; $R^{2}=0.9297$) (**b**) Representative micrographs showing a selection of experimental conditions (positive control (CTRL) and 10 nM and 40 nM Rotenone). Stained against TH (green). Cell nuclei were stained with DAPI (blue). Scale bar = 100 µm. (**c**) Linear regression performed on results of neurite length normalized to the number of TH${}^{+}$-cells and afterwards to positive control. (y=0.9605·x+0.1517; $R^{2}=0.8296$) (**d**) Neurotoxic effects of Rotenone on the neurite network of MDN quantified with manual analysis. Mean neurite length per cell normalized to TH${}^{+}$-cells. (**e**) Linear regression performed on results of neurite length normalized to cell count and afterwards to positive control. (y=0.8460·x+0.2482; $R^{2}=0.7959$) (**f**) showing the neurotoxic effects of Rotenone quantified using the presented neurite outgrowth quantification assay. Mean neurite length per cell normalized to TH${}^{+}$-cells. Data are shown as mean ± SM. * Significantly different (p<0.05) between mean of solvent control (DMSO) and mean of according Rotenone treatment condition. Respectively ** with p<0.01, *** with p<0.001 and **** with p<0.0001.

Further validation of the presented neurite outgrowth quantification assay was conducted using MDN as primary neuronal cells. These neurons grow much longer neurites and develop a higher neurite arborization. To establish different levels of neurite lesioning, we applied Rotenone concentrations ranging from 10 nM to 100 nM. Again, Rotenone treatment resulted in a concentration-dependent effect indicated by a decline in mean neurite length per cell (Figure 5d). Applications of 10 nM and 20 nM resulted in a reduction of neurite length per cell by 21.5±11.5%, respectively, 38.1±2.0%. In total, 40 nM and 100 nM obtained a strong lesion of 48.2±5.1% and 51.9±5.7%. These results were confirmed using the automated approach (10 nM: 22.4±12.0%; 20 nM: 41.7±3.7%; 40 nM: 48.2±6.6%; 100 nM: 56.9±6.4%) (Figure 5f). Conducting linear regression on values of summed neurite length per image shows strong correlation between results from the manual and automated evaluation methods (y=0.3403·x+2109; $R^{2}=0.9297$) (Figure 5a). Linear regression with values normalized to the number of TH${}^{+}$-cells shows even better comparability leading to the following equation: y=0.9605·x+0.1517 ($R^{2}=0.8296$) (Figure 5c). Normalization to DAPI${}^{+}$-nuclei also yields high correlation (y=0.8460·x+0.2482; $R^{2}=0.7959$) (Figure 5e).

## 4. Discussion

We present a fully automated neurite outgrowth quantification assay able for high throughput screening. Our approach was developed using image data generated with differentiated SH-SY5Y cells and MDN and is validated against a manual tracing method. The widely used SH-SY5Y cells and MDN are established in disease modelling of PD [18]. To induce PD pathology, we employed a toxin-based model using the pesticide Rotenone. It inhibits the complex I of the mitochondrial respiratory chain and leads to aggregation of aSYN and induction of oxidative stress through reactive oxygen species [21,22]. The effect of Rotenone toxicity was assessed by quantification of neurite outgrowth and a dose-dependent reduction of neurite length was shown. In addition to neurite length, neurite arborization quantified by our assay also was reduced depending on applied Rotenone concentrations. Concentrations used in this work were in line with other studies that have employed a Rotenone model on similarly differentiated SH-SY5Y cells [50,51] and on MDN [15,52].

Current methods of neurite quantification that are extensively used in toxicological research only enable the assessment of fluorescence images with low neurite density [26,38,39,40]. In our manuscript, we present an algorithm that is capable of high throughput screening for neurite outgrowth also in high density neuronal cultures. Using an algorithmic based evaluation resulted in data acquisition that is independent of human bias and can evaluate large datasets. The pipeline presented in this work, supports centralized adjustment of multiple parameters for every algorithm, making it flexible and adaptable to changes in staining, exposure or magnification and even cell type. This renders the results deterministic and reproducible.

With the implemented skeletonization algorithm from Durix et al. [36], the only parameter needed to adjust was ϵ and skeleton seeding was conducted fully automatically (see Section 2.6 and Section 2.7). This method is designed for discrete shapes and uses a propagation approach to compute a pruned skeleton. Thereby avoiding propagation in directions of noise and the creation of uninformative branches. The rigorous algorithmic foundation and the simple parameter adjustment allowed for the development of an approach which enables the adaption to image data with different morphological characteristics. This is demonstrated by the highly sensitive quantification of neurite outgrowth in the different cell cultures models used in this work. Other skeletonization and pruning methods have to deal with certain drawbacks: They may not preserve the original topology, cannot distinguish between noise and small significant details or rely on unintuitive parameters [36]. The algorithm of Bai et al. [53] was previously used in other bioimaging applications [28,46,54]. However, the approach by Bai et al. relies on a parameter that is mathematically less rigorously defined than the pruning parameter ϵ of the skeleton approach by Durix et al.

Skeletoniziation using ‘thinning’ algorithms is employed by NeuriteTracer [38] and NeurophologyJ [39]. This approach offers high sensitivity by eroding all structures into one-pixel-width skeletons and thereby computes a highly complex skeleton. However, the integration of artefacts and noise is a serious drawback. Thus, this method relies on extensive preprocessing and thereby loses adaptability to different experimental setups. Pani et al. (MorphoNeuroNet [40]) proposed a linear skeletonization using principal curves. However, in preprocessing several smoothing operations have to be implemented due to the susceptibility of generating additional branches on rough edges. The complexity of the algorithm and the requirement of certain image characteristics are typical to this kind of skeletonization.

Correspondingly, our propagated skeleton approach is more robust when dealing with high-density neuron cultures, as compared to previous and less rigorous skeletonization approaches, where it is in general difficult to determine the underlying parameters correctly without user interaction or prior knowledge of the shape that is analyzed. We confirmed the effective quantification of neurite outgrowth by our algorithm allowing for high content screening in neurodegenerative disease models. By examining the skeleton, a more in-depth analysis can be achieved with characterizing different skeleton sections and quantification of the total skeleton area. An assessment of connectivity can be conducted through detecting visual connections between cells and further analyzing the neurite arborization.

## 5. Conclusions

Our automated neurite outgrowth quantification algorithm allows for analysis of dense neurite structures in a high throughput manner and is designed for annotation of two-dimensional immunocytochemical microscopy images of neuronal cultures. We show the successful quantification of neurite outgrowth in large data sets of fluorescently labelled cells. Differentiated neuronal SH-SY5Y cells and MDN treated with different concentrations of Rotenone can be effectively assessed indepently on neurite densitiy. The algorithm shows high comparability with results from manual tracing methods and confirms an effective performance of this method. In addition, the algorithm allows the determination of additional morphological parameters such as cell nuclei, cell somata or neurite branches to perform an in-depth analysis of neuronal cultures. This approach provides a valuable tool for high throughput analysis of neurite outgrowth and allows to avoid time consuming manual assessment.

## Figures and Tables

**Table 1:** Neurite quantification software for high throughput screening of 2D immunocytofluorescence image data.

Name	Degree of Automation	Morphology Measurements	Platform
NeuronJ [26]	semi-automatic	neurite length	ImageJ
Cell Profiler [37]	semi-automatic	neurite length	Python
NeuriteTracer [38]	automatic	neurite length, soma number	ImageJ
NeurophologyJ [39]	automatic	neurite length, soma number and size, neurite attachment points, neurite ending points	ImageJ
MorphoNeuroNet [40]	automatic	neurite length, soma number and size, nucleus number, neurite attachment points, neurite ending points	ImageJ
Omnisphero [28]	automatic	neurite area, neurite length, neurite branching points	Matlab
presented approach	automatic	neurite length, soma number and size, nucleus number and size, neurite ending points, neurite branching points	*C++*, ImageJ

## Data Availability

The data presented in this study are available on request from the corresponding author.

## Abbreviations

The following abbreviations are used in this manuscript:

PD	Parkinson’s Disease
MDN	primary mesencephalic dopaminergic neurons
DMSO	dimethylsulfoxide
PBS	phosphate buffered saline
PBT 1	PBS with 0.1% triton
PBSA	PBT 1 with bovine serum albumin
DAPI	4${}^{\prime }$,6-diamidino-2-phenylindole
SEM	standard error of the mean
ANOVA	analysis of variance
aSYN	α-Synuclein
iPSC	induced pluripotent stem cells
